# Probing Modulation of Attentional Correlates with Aerobic Exercise in Individuals with a History of Concussion

**DOI:** 10.3390/brainsci15080783

**Published:** 2025-07-23

**Authors:** Meghan A. Young, W. Richard Staines

**Affiliations:** Department of Kinesiology and Health Sciences, University of Waterloo, 200 University Ave. E., Waterloo, ON N2L 3G1, Canada; ma4young@uwaterloo.ca

**Keywords:** attention, event-related potentials (ERPs), acute aerobic exercise, performance monitoring, concussion

## Abstract

Background/Objectives: Concussions have been associated with deficits in attentional control. The current work examined whether attentional correlates could be enhanced following acute aerobic exercise in those with a history of concussion (CH). Methods: EEG was collected as participants completed a flanker task to evoke stimulus-locked (N2, P3) and response-locked error-related (ERN, Pe) ERPs, before and after participants completed a bout of acute aerobic exercise at moderate intensity. Conflict was modulated with distance (close/far) and congruency (incongruent/congruent) of the distractors relative to the targets. Results: CH individuals had reduced accuracy in high-conflict conditions, with improvements following exercise. No differences were observed in attentional cognitive control across the four conditions (close/far congruent, close/far incongruent); however, reduced interference control was shown in far conditions, when compared to close conditions. When compared to non-concussed controls, increased accuracy with increased response time in individuals with a concussion history was likely attributed to the speed–accuracy trade-off. Close conditions highlighted a decreased Pe amplitude in CH individuals (as opposed to the active controls), suggesting CH individuals may present with challenges when evaluating an error with working memory. Conclusions: The findings demonstrated acute exercise improved accuracy among CH individuals, and performance monitoring is impacted negatively long term following a concussion.

## 1. Introduction

The most common type of acquired brain injury is concussion [1]. Mild traumatic brain injuries account for the majority, seventy five percent, of annual head trauma cases, and incidence rates can approach twenty percent among Canadian varsity athletes [2,3]. Numerous concussions go unreported to healthcare professionals or go unrecognized [4]. The terms traumatic brain injury and concussion are commonly used interchangeably, with the term mild traumatic brain injury used in reference to a concussion [5,6]. Concussion is often defined as “any transient neurologic dysfunction resulting from a biomechanical force”; however, it often predisposes individuals to long-term consequences in cognitive and motor functions [4,7].

A history of concussion predisposes individuals to a higher risk of subsequent concussions, and exposure to multiple concussions increases the risk of neurodegenerative diseases with aging, namely Alzheimer’s disease [7,8]. There is variability in people, based on their concussion history and other additional factors, whether adults experience cognitive decline or continue to be high functioning throughout aging [4]. Specifically, cognitive reserve is the ability to achieve the same goal without clinical deficit and involves the use of alternate cerebral pathways [4]. Concussed athletes have decreased gray matter volume in the anterior cingulate cortex (ACC) and reduced activation in the dorsolateral prefrontal cortex (DLPFC) [9]. Likewise, chronic impairments in attention, working memory, inhibition and interference control have been shown several years following concussion [10,11,12]. The left frontal regions, areas playing a significant role in executive functions, namely attention, displayed reduced cortical thickness [1].

Event-related potentials (ERPs) give insight into the cognitive processing of stimuli; therefore, EEG is a means to assess electrophysiological disruption following mild traumatic brain injury [13] that may otherwise go undetected [14]. The changes in P3 following mild traumatic brain injury are highly inconsistent with inter-individual variability in spectral frequency activation [15,16]. Nonetheless, amplitude reductions are often visible [14,15]. The P3 (or P300) is an endogenous positive ERP, which can be further categorized into P3a and P3b. The P3a is maximal frontally and represents working memory and attentional mechanisms while processing the task, whereas the P3b is associated with subsequent memory processing and attention in the temporal–parietal region [17]. N2 is a representation of the inhibition of inappropriate motor responses and monitoring responses [4]. Broglio and colleagues [18] demonstrated suppressed P3b and N2 ERPs in response to a three-stimulus oddball task well after concussion (average of 3.4 years). The error-related negativity (ERN or Ne), followed by the error positivity (Pe), is an ERP indicative of conflict monitoring. The ERN plays a role in error feedback and sensitivity to response errors, which is used for improvements in motor outputs thereafter [19]. A linear relationship between ERN reductions and the number of repeated concussions was previously detected; however, it was not visible for Pe amplitude [20]. Pe is the processing of the awareness an error has been made or, inversely, confidence in the correct response, and has been proposed to reflect working memory while evaluating the error [21].

Commonly referred to as a higher-order executive function, selective attention involves deliberate focus, with the allocation of cognitive processes in the midst of surrounding environmental distractors [22]. With attentional control, a number of cortical and subcortical structures are broadly interconnected. Specifically, the prefrontal cortex (PFC), thalamus and ACC are imperative when filtering incoming sensory information and suppressing irrelevant sensory inputs. A hallmark of visual attention is the ability to select relevant information directly related to the goal of the task being completed. The PFC is considered a connection between action selection and perception through attention [23]. Bottom-up (exogenous) attentional control is driven by external factors that are salient, whereas top-down attentional (endogenous) control is internal guidance with the use of prior knowledge and contextual goals [24]. The ERN is generated in the ACC, which is regarded as a component of “error prevention” through the engagement of brain regions. Namely, the DLPFC is essential for the implementation of strategic processing and is recruited for modifying attentional selection efficiency [25]. The flanker task is often used for executive control and selective attention research, where individuals must inhibit or disregard irrelevant stimuli [26]. With easily discriminable targets and flankers, it is proposed that the selection process will be more rapid and less prone to errors [26]. As employed by Danielmeier and colleagues [27] and Wunder & Staines [28], changing the distance between flankers and the target can modulate the degree of conflict. Incongruent stimuli often lead to reduced response speeds and increased error rates, whereas congruent stimuli are often associated with accurate and fast responses [26,29].

Both acute and chronic exercise has neuroprotective effects on aging and presents with various improved neurocognitive functions [29,30]. Hillman and colleagues [29] concluded that physical activity may disrupt cognitive decline throughout aging and lead to improvements in interference control, an aspect of executive control, with physical activity participation in young and middle-aged individuals. An acute bout of aerobic exercise has been shown to enhance activation of the prefrontal cortex, which is associated with improved cognitive performance [30]. A 20 min treadmill walking protocol at 60 percent of heart rate maximum demonstrated improved response accuracy, increased P3 amplitude and improved academic performance compared to rest [31]. Exercise intensity changes P3 amplitude in an inverted U-shaped fashion, thus indicating attentional resources are increased following medium-intensity exercise but decreased following high-intensity pedaling [32]. Chronic exercise, comparing highly aerobically active to sedentary or unfit individuals, is the primary type of aerobic exercise seen in the literature, with minimal research examining the effects of an acute bout of exercise on attention [22,28,29].

Notably, there is a lack of consensus regarding the primary structures affected following mild traumatic brain injury and the magnitude of changes following a concussion. This study aims to further examine the modulation of attentional correlates in CH individuals with a bout of aerobic exercise. Participants completed a 20 min bout of aerobic exercise on a cycle ergometer to allow for a comparison between pre- and post-exercise attentional correlates. It was hypothesized that there would be increased amplitudes in ERPs following an acute bout of aerobic exercise and higher-conflict trials would display a larger amplitude. Based on reductions in P3, N2 and ERN amplitudes seen in concussed individuals in the literature, it was hypothesized that the magnitude of the pre-exercise ERPs would be reduced when compared to their non-concussed counterparts [15,18].

## 2. Materials and Methods

### 2.1. Participants

A total of sixteen previously concussed individuals participated in this study (mean age ± SE: 22.6 ± 0.78, 5 males, 11 females). Participants had to have sustained at least one medically diagnosed concussion with the most recent greater than three months prior to completion, be free of symptomology and be given physical activity participation clearance from a medical provider. Following three months, most cognitive impairments have resolved [33], ensuring a standardized timeline for long-term impairments following concussion. Exclusion criteria included a history of any central or peripheral nervous system diseases or injuries, with mild traumatic brain injury excluded, or substance abuse. Study procedures were approved by the University of Waterloo Research Ethics Board (REB#41931), and all participants provided written informed consent prior to participation.

Participants completed a number of standardized forms, namely the International Physical Activity Questionnaire (IPAQ), Waterloo Health History Questionnaire, General Neurological Questionnaire and the Get Active Questionnaire (GAQ). The IPAQ (2002) allowed for classification of physical activity into moderate and high classifications. The University of Waterloo Health History and General Neurological Questionnaire included information on total number of concussions, time post-concussion, symptoms, previous injuries, headache history, psychiatric history, etc. The GAQ was used as a screening measure to ensure readiness for safe participation in physical activity.

All participants achieved a moderate or high score on the IPAQ (mean METs ± SE: 3398.0 ± 510.7; range: 660–7866), a self-reported questionnaire to assess physical activity participation across numerous domains, including leisure, work, transportation, domestic and gardening activities. A moderate level of physical activity suggests participation in moderate physical activity for 30 min 5 days of the week, 30 min of vigorous-intensity activity per day for at least 3 days or achieving a total of 600 MET minutes each week. A high score on the IPAQ is obtained by either achieving 1500 MET minutes a week with vigorous-intensity activity on a minimum of 3 days or 7 or more days of a combination of walking, moderate- and vigorous-intensity activities to achieve a minimum of 3000 MET minutes a week. Five participants achieved a medium IPAQ classification, and eleven participants achieved a high IPAQ classification. In twelve countries, including Canada, along with individuals aged 18 to 65, the validity of IPAQ has been confirmed [34].

### 2.2. Experimental Design

**Figure 1:**
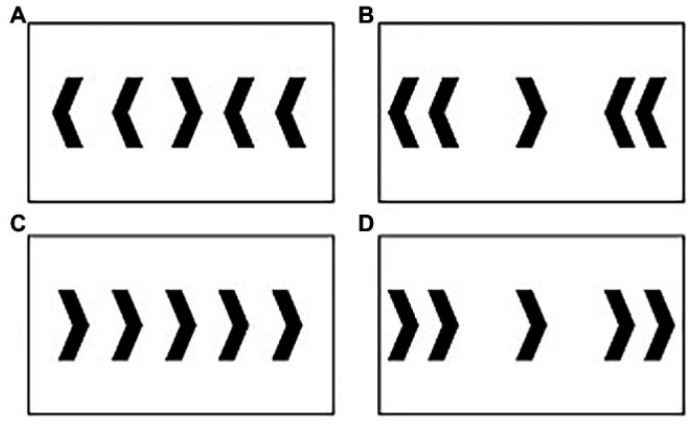
Stimuli used in modified Flanker task: (**A**) close-incongruent; (**B**) far-incongruent; (**C**) close-congruent; and (**D**) far-congruent. Note that although only trials indicating a right target/response are shown, equal proportions of left and right target arrows were presented in the task.

The same speeded modified computer-based flanker task (Figure 1) as employed by Wunder & Staines [28] was completed by participants (STIM2, Compumedics Neuroscan, Charlotte, NC, USA), and the control group, with no history of concussion, was drawn from this study. Specifically, the highly active group from Wunder & Staines [28] served as the control for the concussion history group. This group consisted of 10 healthy, right-handed participants (mean age ± SE: 20.9 ± 0.41), with no reported history of neurological problems. Maximal interference trials occur with close-incongruent, whereas minimal interference occurs during far-congruent trials (Figure 1). Participants were instructed to respond to the direction of the central target arrow while ignoring the four flanking distractor arrows on either side of the target. Participants were asked to respond as quickly and as accurately as possible by clicking the mouse button (left or right) in accordance with the direction of the target arrow. Close and far distractor arrows were either 2.5 or 8.5 cm away from the target. The computer-based flanker task was presented in four blocks of 200 trials with a total of 800 trials, 80 percent of which were congruent and 20 percent incongruent, with 50 percent close and 50 percent far. This totals 310 close-congruent, 310 far-congruent, 90 close-incongruent, and 90 far-incongruent trials. Carter and colleagues [25] demonstrated that incorporating 80 percent of congruent trials induced more top-down control and increased conflict with incongruent trials in the ACC. Stimuli were displayed for 200 ms with an interstimulus interval randomized between 1200 and 1400 ms.

### 2.3. Acute Aerobic Exercise Intervention

Following the modified computer-based flanker task, participants partook in an exercise intervention on a stationary bike. Resting heart rate was determined before exercise. To maintain mechanical efficiency across all participants, pedaling rate was between 50 and 70 revolutions per minute. A 20 min bout of acute aerobic exercise, invoking a 60 percent maximum heart rate, as employed by Popovich & Staines [22] and Hillman et al. [31], was completed. Percentage of maximum heart rate was calculated using the equation 220-age [35]. The ergometer resistance level was always adjusted to a minimum of level 1 for each participant. Heart rate was monitored with a Polar heart rate monitor, and the 10-Point Borg Scale was used as the measure of Rate of Perceived Exertion (RPE) every 5 min. Precautions were taken throughout completion of the exercise intervention, in case termination of exercise was necessary. Participants rested for approximately 5–10 min following the acute bout of exercise until heart rate was within 10% of the original resting heart rate. Next, the computer-based flanker task was completed again with a total of 800 trials.

### 2.4. Electroencephalography Data Collection

EEG data were recorded from 15 electrode sites (FP1, FZ, FCZ, CZ, CPZ, PZ, OZ, F3, F4, FC3, FC4, C3, C4, P3, P4) referenced to the linked mastoids, using a 32-channel Quik-Cap (Neuroscan, Compumedics, Charlotte, NC, USA) in accordance with the International 10–20 System for electrode placement. Impedance was less than 5 kohms for each electrode site, and EEG data were filtered (DC-100 Hz) and digitized (500 Hz, SynAmpsRT, Curry, Compumedics Neuroscan) prior to being saved for offline analysis.

Continuous data were then epoched to extract response- or stimulus-locked ERPs. Artifact detection was conducted on the full epoch, and epochs were excluded if the voltage threshold of ±100 µV was exceeded. Each epoch was also manually inspected, and trials with noticeable artifacts (e.g., eye blinks, muscle contractions, eye movements) were eliminated before averaging. There were 310 close-congruent, 310 far-congruent, 90 close-incongruent, and 90 far-incongruent trials collected per participant, and after contaminated trials were eliminated, the final trace for each experimental condition consisted of 284 (congruent) and 70 (incongruent) artifact-free epochs on average. Stimulus-locked epochs were taken from 100 ms pre-stimulus to 600 ms post-stimulus and were used to extract the P3 and N2 ERP peaks [36]. The P3 was the maximal positive deflection extracted between 300 and 500 ms from central electrode sites, maximal at CZ, and was measured separately for the far-congruent, far-incongruent, close-congruent, close-incongruent stimuli to further understand attentional resource allocation for varying conditions. The N2 was the maximal negative peak between 200 and 350 ms in the difference trace obtained from subtracting congruent from incongruent trial epochs. The N2 from far trials was differentiated from close trials to modulate levels of conflict between trials [28] and was measured from frontocentral electrodes, maximal at FCZ, consistent with Groom and Cragg [36].

Response-locked epochs were extracted between 100 ms pre- and 600 ms post-response. FCZ was the electrode site from which both the ERN and Pe were extracted, consistent with previous studies [19]. The ERN was extracted by subtracting correct from incorrect trial epochs and detecting the negative peak occurring between 0 and 150 ms post-response, with far and close trials measured separately. Pe was the maximum peak between 100 and 300 ms, with far and close trials measured separately [28].

### 2.5. Data Analysis

For the analysis of ERP peaks (Pe, ERN, N2) and response time/accuracy, a two-way repeated measures ANOVA with factors TIME (pre-, post-exercise) and CONFLICT (close, far) was used. For the analysis of the P3 ERP peak, a two-way repeated measures ANOVA with the factors TIME (pre-, post-exercise) and CONFLICT (close-congruent, close-incongruent, far-congruent, far-incongruent) was used. *Post hoc* Tukey analyses were used to follow-up any significant effects from the ANOVAs. An independent means sample *t*-test was used to compare the highly active (HA) group ERPs from Wunder & Staines [28] to pre-exercise ERPs of the individuals with a concussion history. The HA group was composed of 10 participants (mean age ± SE: 20.9 ± 0.41, three male) and underwent the same speeded modified computer-based flanker task following high IPAQ questionnaire classification [28]. Prior to computation of the ANOVAs, residual errors were plotted and inspected to ensure that the assumptions for normality and homogeneity of variance were met. Statistical analyses were completed using SAS 9.0, and an alpha value of 0.05 was used to define significance.

## 3. Results

### 3.1. Participant and Behavioural Data

Most participants sustained one or two concussions, aside from two participants who were diagnosed with three and twelve concussions, respectively (mean number of concussions ± SE: 2.13 ± 0.68). The timeline since the last date of concussion greatly varied among participants (mean time since last concussion ± SE: 6.8 ± 0.74 years), with an approximate mean of 7 years post-concussion. Similarly, the longest symptom duration was anywhere from days and weeks to months and years. The mechanism of concussion was often related to sports participation in hockey, football, rugby, soccer, basketball, and snowboarding.

**Figure 2:**
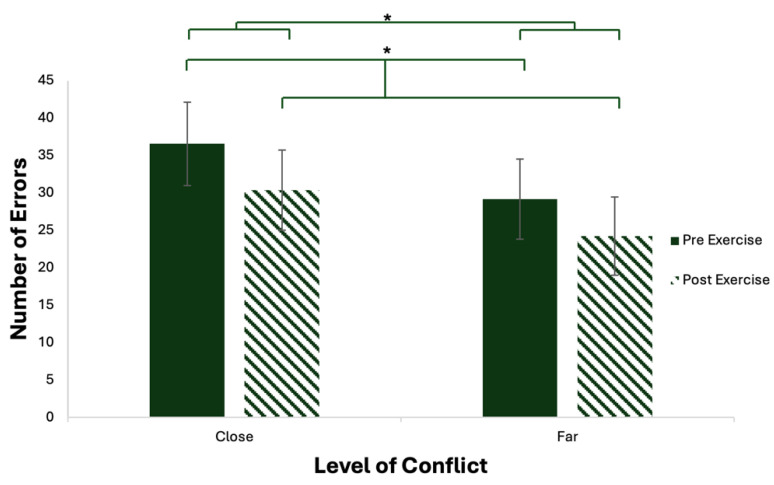
Mean number of errors made in close and far conflict, pre-exercise and post-exercise; error bars denote ± standard error (SE); * denotes *p* < 0.05.

A two-way repeated measures ANOVA revealed a significant effect of time (F_1,15_ = 6.73, *p* = 0.02, Ƞ_p_^2^ = 0.218) on accuracy in CH individuals. As shown in Figure 2, pre-exercise had a greater number of errors, when compared to post-exercise number of errors. A main effect of conflict on number of errors was also observed (F_1,15_ = 24.35, *p* < 0.01, Ƞ_p_^2^ = 0.291). Specifically, close conditions showed a higher number of errors compared to far conditions. There was no interaction between time and conflict (F_1,15_ = 0.42, *p* = 0.53, Ƞ_p_^2^ = 0.039).

When looking at response time, there was no significant effect of time (F_1,15_ = 2.88, *p* = 0.11, Ƞ_p_^2^ = 0.087), conflict (F_1,15_ = 3.37, *p* = 0.09, Ƞ_p_^2^ = 0.075) or interaction between time and conflict (F_1,15_ = 2.30, *p* = 0.15, Ƞ_p_^2^ = 0.022). Although not significant, distractors with higher conflict (close) showed a higher mean response time than those with lower conflict (far) (mean close ± SE: 347.57 ± 42.88 ms; mean far ± SE: 337.86 ± 46.09 ms).

### 3.2. Event-Related Potentials (ERPs)

#### 3.2.1. P3

**Figure 3:**
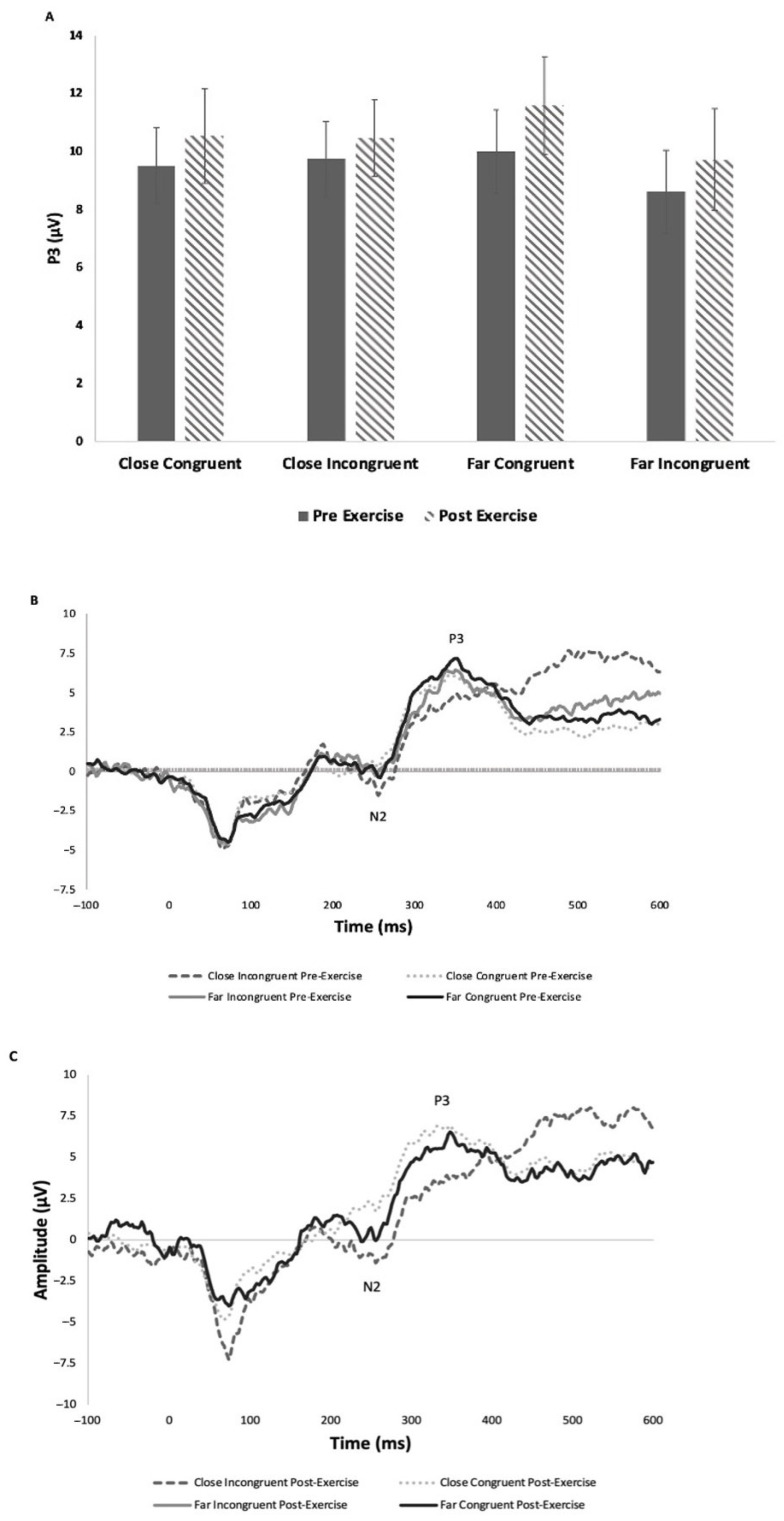
(**A**) Mean (± SE) P3 amplitude from CZ electrode site; (**B**) grand average stimulus-locked waveform, P3 pre-exercise from CZ electrode site; (**C**) grand average stimulus-locked waveform, P3 post-exercise from CZ electrode site.

P3 was measured from the CZ electrode site across all participants. There were no significant effects on P3 amplitude across conflict (F_3,45_ = 1.36, *p* = 0.27, Ƞ_p_^2^ = 0.027), time (F_1,15_ = 0.49, *p* = 0.50, Ƞ_p_^2^ = 0.014) or interaction between time and conflict (F_3,45_ = 0.25, *p* = 0.86, Ƞ_p_^2^ = 0.004) (Figure 3). There was a significant main effect of conflict on P3 latency (F_3,45_ = 9.87, *p* < 0.001, Ƞ_p_^2^ = 0.179), and Tukey comparisons revealed that P3 latency was significantly longer in the CI condition (mean ± SD: 422.4 ± 69.5 ms) than all of the other three (mean ± S D: FC—366.5 ± 66.9 ms; FI—375.8 ± 67.7 ms; CC—368.8 ± 69.2 ms).

#### 3.2.2. N2

**Figure 4:**
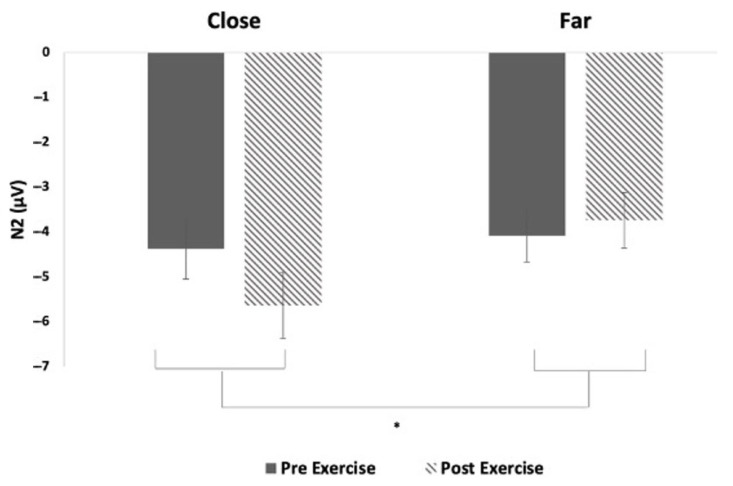
Mean (± SE) N2 amplitude from FCZ electrode site; * denotes *p* < 0.05.

N2 was measured from the FCZ electrode site across all participants. There was a significant effect of the level of conflict on N2 amplitude (F_1,15_ = 4.83, *p* = 0.04, Ƞ_p_^2^ = 0.084; Figure 4). There was no significant effect of time (F_1,15_ = 0.51, *p* = 0.49, Ƞ_p_^2^ = 0.012), nor was there a significant interaction between time and conflict (F_1,15_ = 2.99, *p* = 0.10, Ƞ_p_^2^ = 0.047). There were no significant main effects or interactions on N2 latency (overall mean latency 283.3 ms).

#### 3.2.3. Error-Related Negativity (ERN)

**Figure 5:**
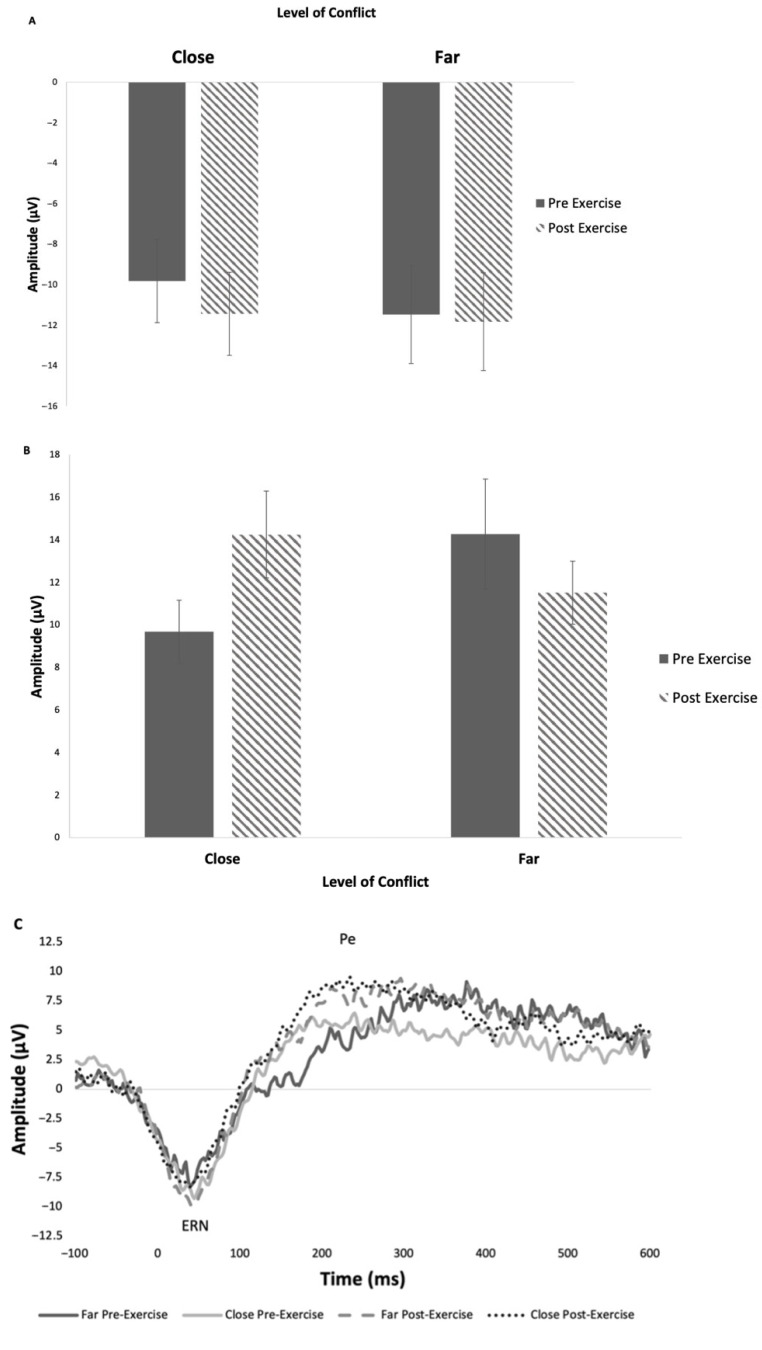
(**A**) Mean (± SE) ERN amplitude from FCZ electrode site; (**B**) mean (± SE) Pe amplitude from FCZ electrode site; (**C**) grand average stimulus-locked waveform Pe and ERN pre-exercise and post-exercise from FCZ electrode site; difference between correct and incorrect trials.

ERN was measured from the FCZ electrode site across all participants (Figure 5). There were no significant effects of time (F_1,15_ = 0.25, *p* = 0.63, Ƞ_p_^2^ = 0.009) or conflict (F_1,15_ = 0.70, *p* = 0.42, Ƞ_p_^2^ = 0.009) on ERN amplitude. Similarly, there were no significant interactions between time and conflict (F_1,15_ = 0.22, *p* = 0.65, Ƞ_p_^2^ = 0.004). There were no significant main effects or interactions on ERN latency (overall mean latency 42.3 ms).

#### 3.2.4. Error Positivity (Pe)

Pe was measured from the FCZ electrode site across all participants. There was no significant effect of time (F_1,45_ = 0.36, *p* = 0.55, Ƞ_p_^2^ = 0.008) or conflict (F_1,45_ = 0.38, *p* = 0.54, Ƞ_p_^2^ = 0.008). There was a significant interaction effect between time and conflict (F_1,45_ = 5.84, *p* = 0.02, Ƞ_p_^2^ = 0.115; Figure 5B); however, Tukey comparisons revealed no significant pairwise differences. Pre-exercise close conflict showed a reduced amplitude compared to pre-exercise far. The inverse is true following exercise, with close eliciting a higher amplitude. There were no significant main effects or interactions on Pe latency (overall mean latency 223.9 ms).

### 3.3. Concussion History vs. Non-Concussed Highly Active Controls

#### 3.3.1. Behavioral Data

**Figure 6:**
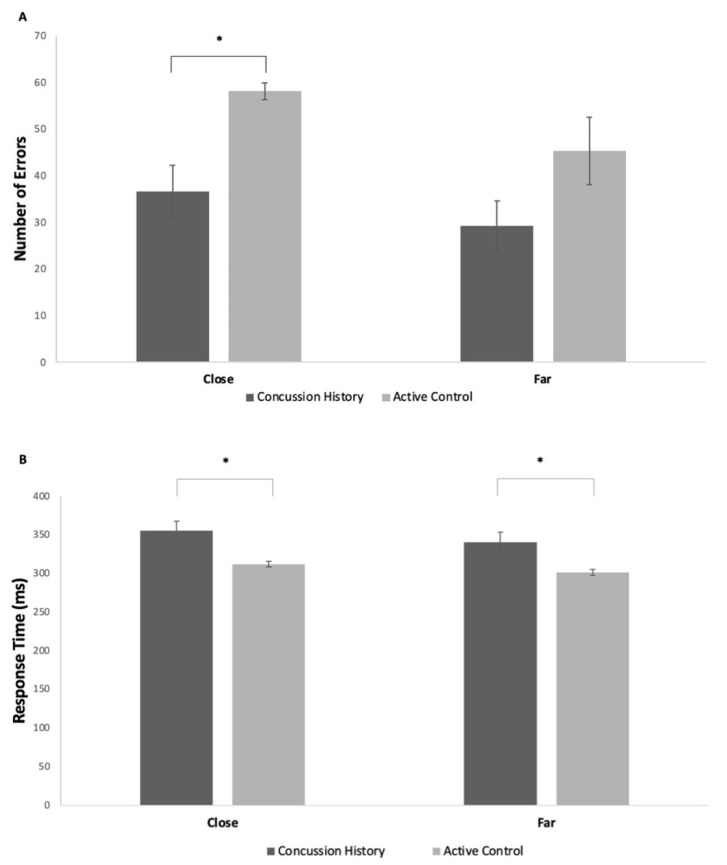
(**A**) Mean (± SE) number of errors made in close and far conflict between participants with a concussion history and active controls; (**B**) mean (± SE) response time in close and far conflict between CH participants and active controls; * denotes *p* < 0.05.

Active controls had an increased number of errors in the close condition when compared to CH individuals (*p* = 0.04; Figure 6A). There was also a statistically significant difference in response time between individuals with a concussion history and active controls in close (*p* = 0.02) and far conditions (*p* = 0.03), where CH individuals were slower in responding (Figure 6B).

#### 3.3.2. Stimulus-Locked ERPs

There were no significant differences between P3 pre-exercise for CH individuals across the conditions (*p* > 0.05) (close-congruent (*p* = 0.13), close-incongruent (*p* = 0.95), far-congruent (*p* = 0.07) and far-incongruent (*p* = 0.29)) when compared with the highly active group in Wunder & Staines [28]. Similarly, the N2 amplitude between CH participants and the active control group revealed no statistically significant difference between the close (*p* = 0.10) and far conditions (*p* = 0.25).

#### 3.3.3. Response-Locked ERPs

**Figure 7:**
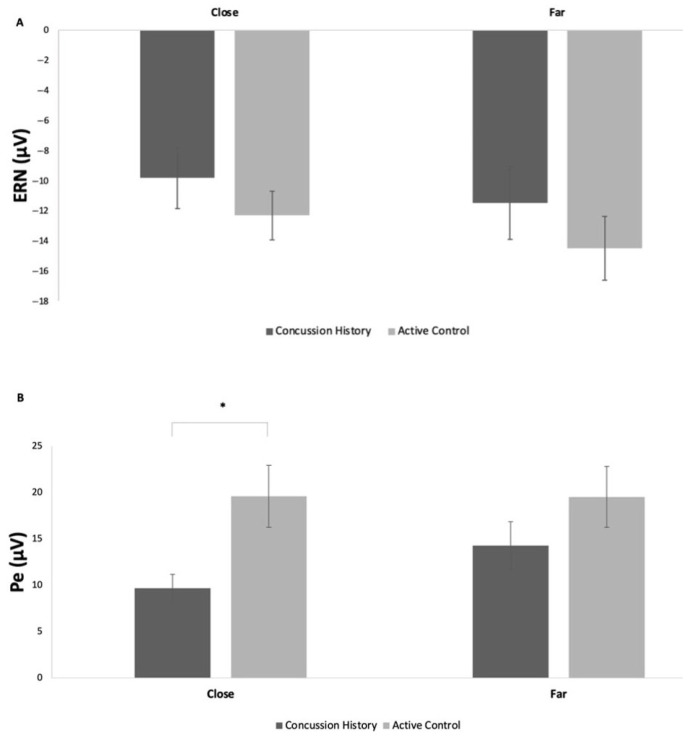
(**A**) Mean (± SE) ERN amplitude in concussion history participants compared to the active group (control) in Wunder & Staines (2022); (**B**) mean (± SE) Pe amplitude in participants with a concussion history compared to the active group (control) in Wunder & Staines (28); * denotes *p* < 0.05.

For response-locked ERPs, the comparison between the CH pre-exercise ERN amplitude and controls demonstrated no statistically significant difference in the close (*p* = 0.40) and far (*p* = 0.40) conflict conditions between the two groups (Figure 7A). However, the Pe amplitude was significantly smaller in the close condition for Pe amplitude in CH versus non-concussed controls (*p* = 0.01, Figure 7B) but not in the far condition (*p* = 0.24).

## 4. Discussion

Participants with a concussion history (CH) had improved accuracy following an acute bout of exercise. Additionally, CH individuals displayed an increased number of errors in the higher-conflict (close) conditions, compared to the lower-conflict (far) conditions. Similar trends were visible in response time, with improvements following exercise and higher-conflict conditions eliciting increased response times. No significant differences were observed with the acute exercise bout in P3 across the four conditions or in the ERN or Pe in the close and far conditions. CH individuals were able to differentiate level of interference control, as demonstrated by the statistical difference in conflict among N2 amplitudes. When compared with active controls, CH participants committed a reduced number of errors but had a more prolonged response time in both close and far conditions. The close condition demonstrated a reduction in Pe amplitude in CH individuals, suggesting impairments in working memory or error awareness.

### 4.1. Participants with a Concussion History

In the present investigation, there was a statistically significant effect of time and conflict on accuracy. There was a reduction in the number of errors committed following exercise in the CH participants. In addition, as expected, the CH individuals had decreased accuracy in the higher-conflict conditions (close), as opposed to the lower-conflict (far) conditions. It is worth noting that Hillman and colleagues [31] discerned a greater accuracy with increased cardiorespiratory fitness in healthy individuals. Wunder & Staines [28] discussed the ability to better discriminate between targets and manage interference in the highly active group, as displayed by the increased amplitude in N2 in close as opposed to far trials. The low active group did not have a different response between high- and low-conflict trials. These individuals with a history of concussion recruited in the current study fell under high and moderate IPAQ classifications, and, therefore, cardiorespiratory fitness may have an influence. Overall, active CH individuals are able to adjust their responses according to the level of interference, and an acute bout of aerobic exercise leads to improvements in accuracy (Figure 2).

No significant differences were recorded when comparing pre-exercise and post-exercise response times. Hillman et al. [31,37] found no differences in response time following acute exercise and concluded that the response time is insensitive to an acute bout of aerobic exercise. Predictably, a close level of conflict resulted in increased response times, by a mean of 10 ms, in comparison to the far levels of conflict. This further confirms that the previously concussed participants were able to modulate the level of interference, with increased time in higher-conflict conditions and reduced time in lower-conflict conditions.

This study’s primary objective was to determine the differences in neural markers induced by an acute bout of exercise in individuals with a concussion history as a result of short-term excitability changes. In contrast to the prior literature, there were no differences observed in P3 amplitude across the four conditions: close-congruent, close-incongruent, far-congruent and far-incongruent. Hillman and colleagues [31] demonstrated that an acute bout of exercise on a treadmill increased P3 amplitude for incongruent trials in comparison to rest. In accordance with the primary hypothesis, as shown in Figure 3, there were modest, non-significant increases in P3 amplitude following an acute bout of exercise, with no differences in amplitudes associated with the level of conflict. These results suggest that these CH participants did not display differences in attentional cognitive control. De Beaumont and colleagues [15] found P3 amplitude suppression in multi-concussed athletes, which did not occur in the singly concussed athletes. This could be a factor for the negligible differences in P3 amplitudes across individuals, with a mean number of 2.1 concussions sustained. Research should control for the number of concussions and continue to examine differences in attentional mechanisms following an acute bout of exercise as there may be benefits of increased attention following exercise. Furthermore, Hillman et al. [31] observed the exercise-induced modulation in P3 25 min after exercise cessation. Further research needs to inspect the duration of exercise-induced benefits and the magnitude of the cognitive function enhancements, as well as the breakdown of components of P3a and P3b amplitudes.

With reference to N2 amplitude, there was a significant difference between close and far conflicts. Distinctively, the level of interference control was processed significantly differently in higher-conflict (close) trials compared to lower-conflict (far) trials. Moreover, Danielmeier et al. [27] modulated the distance between flanking and target arrows to alter the degree of interference, allowing them to track variations in N2 amplitude. In a comparable manner, the magnitude of N2 appeared highest in a flanker task when a congruent trial came before an incongruent trial [32]. When inspecting the highly active group in Wunder & Staines [28], the N2 amplitude was significantly higher in the close (higher conflict) condition. The differences are more similarly depicted in the CH individuals following the acute bout of aerobic exercise. These results suggest that there may be a difference in the interference control and endogenous processing of conflict before and after completing an acute bout of aerobic exercise in CH individuals. To conclude, an increased amount of interference control is present in the high-conflict trials, which is illustrated by the increased N2 amplitude in close conditions.

The extraction of ERPs during performance monitoring included both the ERN and Pe. The ERN demonstrated no significant effect of exercise. According to Krigolson et al. [19], the ERN is confined to the medial frontal cortex, namely the anterior cingulate cortex (ACC), and it interacts directly with the basal ganglia. ERN amplitudes in individuals with a history of concussion were similar pre- and post-exercise across conditions, and they did not differ from controls. According to Gehring and Willoughby [38], ERN is elicited by “the appraisal of the penalty”. The penalty between clicking left or right on the mouse may not be consequential enough to demonstrate pronounced differences in amplitudes. De Beaumont et al. [39] and Pontifex et al. [20] discussed the negative correlation between the number of concussions and ERN amplitude suppression. Potentially, ERN is only sensitive to the long-term effects of numerous concussive events, as proposed for the P3 amplitude [20,40]. Additionally, it would be sensible to examine post-error response slowing and to adjust feedback salience in future studies to understand whether these participants employ top-down attentional control in subsequent trials in an attempt to improve performance [40].

Similarly, there were no significant differences in Pe amplitudes across the conditions or time, with a slight observable increase in amplitude following the acute bout of aerobic exercise. However, in contrast to the ERN, there was an interaction between time and conflict, where the Pe amplitude increased post-exercise for low-conflict and decreased post-exercise for high-conflict conditions, suggesting that exercise has a different effect across levels of conflict (Figure 5B). Aligned with Pontifex et al. [20], Pe amplitude does not seem aligned with chronic impairments observed in mTBI groups. In discordance with hypothesis 1, pre-exercise disconfirmed that higher-conflict trials will display a higher amplitude; however, following exercise, the close conditions generated a higher Pe amplitude compared to the far conditions. The acute bout of exercise seems to elicit the expected response of an increased amplitude in higher-conflict trials, as observed in the highly active group from Wunder & Staines [28].

### 4.2. Comparison Between Concussion History and Active Controls

When inspecting behavioral data, it is surprising that the CH group exhibited a reduced number of errors in both the close and far conditions, compared to the active controls. Statistical significance was achieved in the close condition (higher level of conflict), with a similar trend visible in the far condition (lower level of conflict). Pontifex et al. [41] looked at the distribution of commission errors, which are incorrect responses and omission errors, which are nonresponses. The mTBI group had an increased number of omission errors but no group differences for commission errors, which may be related to the inability to maintain attentional vigilance. Potentially, these differences may be replicated with omission errors in flanker task performance through the further categorization of errors; however, the active group did commit an increased number of errors in both close and far conditions. Another factor for consideration is that CH participants may be using cognitive reserve through alternate cerebral pathways to complete the same task.

Along with the reduction in errors, the CH group also responded more slowly compared to the active controls. Presumably, the trade-off between accuracy and speed must be considered. The previously concussed individuals were more accurate in their response due to the increased response time; therefore, emphasis was placed on accuracy. It has been shown that CH response time deficits generally subside long term [42]. In the present study, CH participants displayed prolonged response times that were greatest in the highest-conflict condition. Interestingly, the P3 latency was also significantly longer in this condition. Potentially, the number of prior concussions plays a role in prolonged response time. A debriefing interview may allude to the components stressed by the participant in the completion of the task to further understand the results in subsequent research.

Pontifex et al. [41] demonstrated a linear relationship between ERN reductions and the number of repeated concussions; however, this was not seen in Pe. In both close and far conditions, in accordance with the second hypothesis, the active control group had increased ERN amplitudes. The results suggest CH individuals may experience challenges in differentiating the expected versus the actual response.

Following the mismatch between the incorrect execution and correct motor plan facilitated by ERN, Pe processes the conscious evaluation [39]. In the present study, a statistically significant reduction in Pe amplitude occurred in CH individuals (in comparison to active controls). This has not previously been depicted in the literature. It is probable that CH individuals have more difficulty with the manipulation of working memory during the evaluation of an error in close conditions. The far conditions may not elicit enough conflict to facilitate difficult planning and problem solving in previously concussed individuals. Regardless, the present study’s results suggest alterations in performance monitoring are present long term following a concussion.

### 4.3. Potential Mechanisms

A growing body of evidence has brought attention to the numerous benefits of aerobic exercise on physical and cognitive functions. Aerobic exercise has been shown to help mitigate the risk of cognitive decline associated with aging and enhancements in attention, executive function and memory [29,30]. Interestingly, areas involved in executive control, such as the prefrontal cortex, may be amenable to an exercise intervention [16]. Yanagisawa and colleagues [30] demonstrated that an acute bout of moderate exercise has the ability to improve interference control and cognitive performance with increased activation of the DLPFC. There are a number of mechanisms suggested to account for the underlying changes following an acute bout of aerobic exercise. It has been suggested that aerobic exercise may enhance arousal in an inverted U-shaped fashion [32]. Additionally, MacIntosh and colleagues [43] found acute exercise induces alterations in cerebral blood flow. Aerobic exercise results in the upregulation of key neurochemicals important for synaptic plasticity [16]. Altogether, there are a number of potential mechanisms which may account for exercise-induced alterations in behavioral data and ERPs.

### 4.4. Limitations

Broglio and colleagues [18] discussed the underreporting that often occurs with concussion or concussive symptomology. Moore et al. [44] raised the concern that individuals exposed to repetitive head impacts without concussive symptoms may be predisposed to the same long-term consequences that previously concussed individuals experience. Future research should look to differentiate the severity, number of concussive events, timelines of concussive symptoms experienced, the potential for sub-concussive events, and mechanism of concussion to allow further comparisons. Although the IPAQ is considered reliable, it is a self-reported questionnaire that is subject to memory bias and/or social desirability bias, resulting in overreporting. Future studies could incorporate a more objective fitness measure such as maximal oxygen uptake in addition to the IPAQ, as well as having the control (non-concussed) group perform the experimental tasks both before and after exercise. Lastly, a limitation relates to the large heterogeneity among participants. All participants took part in different types of physical activity, and one individual sustained 12 concussions, compared to the mean of 2.1 concussions. However, close attention was given to this participant when performing analyses, and their data were not an outlier to the group on any of the measures. Most of the participants were female and of white ethnicity; therefore, generalizing the conclusions among all ethnicities and sexes may not be applicable.

## 5. Conclusions

This study contributes to the limited literature on an acute bout of aerobic exercise in individuals with a concussion history and the comparison of long-term changes in behavioral data, as well as neuroelectric measures between those with a history of concussion and their non-concussed counterparts. Performance monitoring suffers long-term consequences in individuals with a concussion history. CH individuals exhibit differences in interference control across levels of conflict, as depicted with N2. Acute exercise increased accuracy in CH patients and modulated neural markers of performance monitoring. Further research should continue to employ a bout of aerobic exercise in CH individuals to understand the modulation of attentional correlates and the plausible benefits of aerobic exercise to be integrated into cognitive rehabilitation protocols.

## Data Availability

The datasets generated for this study are available on request from the corresponding author.
